# Improved HYDROPS: Imaging of Endolymphatic Hydrops after Intravenous Administration of Gadolinium

**DOI:** 10.2463/mrms.tn.2016-0126

**Published:** 2017-05-22

**Authors:** Shinji Naganawa, Hisashi Kawai, Toshiaki Taoka, Michihiko Sone

**Affiliations:** 1 Department of Radiology, Nagoya University Graduate School of Medicine, 65 Tsurumai-cho, Shouwa-ku, Nagoya, Aichi 466-8550, Japan; 2 Department of Otorhinolaryngology, Nagoya University Graduate School of Medicine, Aichi, Japan

**Keywords:** magnetic resonance imaging, endolymphatic hydrops, temporal bone disease, fluid attenuated inversion recovery

## Abstract

To improve the imaging protocol for the evaluation of endolymphatic hydrops after intravenous administration of a gadolinium-based contrast agent, we modified our previously reported hybrid of reversed image of positive endolymph signal and native image of positive perilymph signal (HYDROPS) method. Although the scan time of the new protocol was half that of the previous one, there were no significant differences between two protocols in the mean contrast noise ratio between the endolymph and perilymph and the area ratio of the endolymph size values in nine patients.

## Introduction

Clinical evaluation of endolymphatic hydrops (EH) using magnetic resonance (MR) imaging was firstly performed in patients with suspected Ménière’s disease at 24 hours after intratympanic administration of gadolinium-based contrast agent (IT-GBCA).^1^ However, due to the invasiveness of IT-GBCA, MR imaging at 4 hours after intravenous administration of a single dose of gadolinium-based contrast agent (IV-SD-GBCA) has become increasingly popular in clinical practice.^1^

To simulate the image contrast of the three dimensional (3D)-real inversion recovery (IR) images usually used for the IT-GBCA method, the subtraction of two images obtained with different inversion times - *HYbriD of Reversed image Of Positive endolymph signal and native image of positive perilymph Signal* (HYDROPS) images - has been employed for examination by IV-SD-GBCA.^1^

A HYDROPS image is the subtraction of a positive endolymph image (PEI) from a heavily T_2_-weighted 3D-fluid attenuated inversion recovery (hT_2_w-3D-FLAIR) or positive perilymph image (PPI). The hT_2_w-3D-FLAIR image is quite sensitive to low concentrations of GBCA in fluid.^2^ Due to the low signal to noise ratio, we need approximately 30 minutes of scan time to obtain a typical HYDROPS image.^3^ If we could shorten the scan time, we could decrease the chance of patient motion and increase the throughput of the MR examination. On FLAIR images, the signal of non-attenuated substances increases by increasing the repetition time.^4,5^ To further increase the sensitivity of the hT_2_w-3D-FLAIR acquisition to low concentrations of GBCA, we increased the repetition time (TR). Furthermore, by increasing the TR, it was possible to increase the refocusing flip angle in the regulated specific absorption rate. An increased refocusing flip angle can contribute to an increase in the signal to noise ratio of long T_2_ substances, such as fluid.^6^ The purpose of this study was to compare the contrast-noise ratio (CNR) between the endolymph and perilymph on HYDROPS images obtained with a conventional protocol to that obtained with the newly proposed and improved HYDROPS protocol (i-HYDROPS), and to evaluate the feasibility of the improved protocol. We also compared the area ratio of the endolymphatic space to the total lymphatic fluid space (% endolymphatic space [%EL]) between the images obtained with the two protocols.

## Materials and Methods

### Patients

Eighteen ears in nine patients with a clinical suspicion of endolymphatic hydrops were included. Patients underwent an MR examination for the evaluation of endolymphatic hydrops. The shorter scan time protocol had been added to the conventional protocol for a backup in case of patients’ motion during the longer scan time. Experienced otorhinolaryngologists determined the indication for the MR examination. The patients included four men and five women with an age range of 23–73 years (median 57). The medical ethics committee at our institution approved this retrospective study with a waiver of informed consent.

### MR imaging

All MR imaging was performed using a 3-tesla scanner (Verio, Siemens, Erlangen, Germany) with a 32-channel array head coil. MR scanning was performed 4 hours after IV-SD-GBCA (0.2 ml/kg body weight or 0.1 mmol/kg body weight) of gadolinium diethylenetriaminepentaacetic acid bismethylamide (Gd-DTPA-BMA) (Omniscan, Daiichi-Sankyo Pharm, Tokyo, Japan). All patients had an estimated glomerular filtration rate (eGFR) value exceeding 60 mL/min/1.73 m^2^.

According to the clinical protocol used by the hospital for the evaluation of endolymphatic hydrops,^6–8^ the patients underwent heavily T_2_-weighted MR cisternography (MRC) for an anatomical reference of the total lymph fluid, a hT_2_w-3D-FLAIR scan with a 9000 msec TR, and a 2250-msec inversion time (positive perilymph image [PPI]), and a positive endolymph image (PEI) with the same TR, and a 2050-msec inversion time at 4 hours after the administration of the IV-SD-GBCA. The maximum refocusing flip angle in the echo train was 120 degrees.^7,8^ Parameters were set as previously reported.^6,9^ The i-HYDROPS image with a shorter scan time was obtained as a backup for the conventional protocol. Detailed scan parameters are listed in Table 1. Briefly, the shorter protocol used a longer TR of 16000 msec and a higher refocusing flip angle of 180 degrees both for the PPI and the PEI. A TR of 16000 msec is the maximum value possible on the scanner. The scan time for the HYDROPS image was 28.6 minutes and that for the i-HYDROPS image was 14 minutes in total. To achieve a shorter scan time, the number of excitations was reduced from 4 to 1.4 and the slice oversampling rate from 38.5% to 7.7% for the i-HYDROPS image. The new scan protocol was optimized and tested as part of another volunteer study, although the details of the protocol have not been described previously.^10^

### Image processing

The image processing methods were similar to those used in the previous study.^7^ Briefly, the HYDROPS image was generated by a subtraction of the PEI from the PPI and the i-HYDROPS image was generated by a subtraction of the PEI from the PPI obtained with the new protocol. For the subtraction, negative signal values were allowed. During this step, no image registration program was applied.

### Image analysis

A neuroradiologist with 28 years of experience in MR imaging performed the image analysis. For the region of interest (ROI) of the perilymph signal, the scala tympani in the basal turn of cochlea was manually contoured on both the HYDROPS and the i-HYDROPS images on a PACS viewer (Rapid-Eye, Toshiba Medical Systems, Tokyo, Japan). For the ROI of the endolymph, the upper part of the utricle was contoured on both the HYDROPS and the i-HYDROPS images. The signal intensities of the right and the left sides were averaged. A noise ROI was defined in the air area of the lower right corner of the image at an identical location on both images. Details of the ROI placement were described previously.^10^ The CNR was defined as follows:

$$\begin{array}{ll} \text{CNR}= & (\text{Signal}\;\text{intensity}\;\text{value}\;\text{of}\;\text{the}\;\text{perilymph}\;\text{ROI}-\\ & \text{Signal}\;\text{intensity}\;\text{value}\;\text{of}\;\text{the}\;\text{endolymph}\;\text{ROI})\\ & /\text{standard}\;\text{deviation}\;\text{of}\;\text{the}\;\text{noise}\;\text{ROI} \end{array}$$

Mean CNR values were compared between the HYDROPS and the i-HYDROPS images using a Student’s *t*-test.

The area ratio of the endolymph area to the total lymph area (%EL) was measured using a previously reported method for the bilateral cochlea and the vestibule in the nine patients on both the HYDROPS and the i-HYDROPS images using OsiriX software, version 5.6 (freely downloadable at http://www.osirix-viewer.com/) on an iMac computer (Apple Computer Inc, Cupertino, CA, USA) respectively.^7^ Pixels with negative values were estimated as endolymph and those with positive values were estimated as perilymph. The %EL values were compared between the HYDROPS and the i-HYDROPS images both for the cochlea and the vestibule of the 18 ears. The %EL values obtained by the two protocols were compared by Student’s *t*-test and a Pearson’s correlation coefficient was calculated. Statistical analyses were performed using R software, version 3.2.2 (freely downloadable at https://www.r-project.org/).

## Results

**Fig 1.**
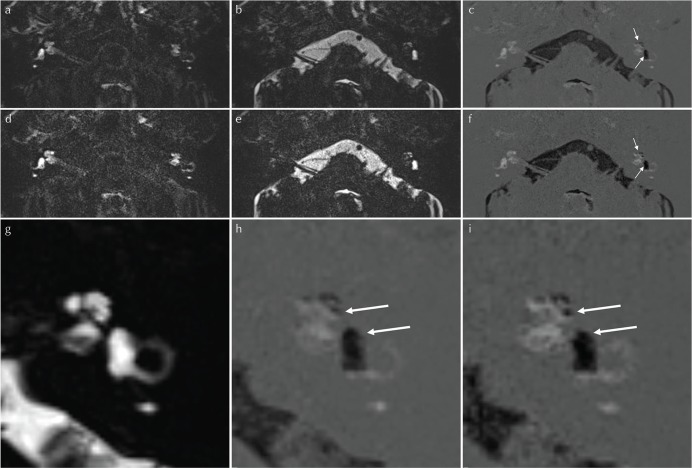
Representative images from a 57-year-old female patient with significant endolymphatic hydrops in both the cochlea and the vestibule on the left side. (**a**) conventional positive perilymph image (PPI) (TR/TE/TI = 9000/544/2250, all in milliseconds), (**b**) conventional positive endolymph image (PEI) (repetition time [TR]/echo time [TE]/inversion delay [TI] = 9000/544/2050) and (**c**) hybrid of reversed image of positive endolymph signal and native image of positive perilymph signal (HYDROPS) image, (**d**) improved PPI (TR/TE/TI = 16000/544/2900), (**e**) improved PEI (TR/TE/TI = 16000/544/2500) and (**f**) improved HYDROPS protocol (i-HYDROPS) image. The signal magnitude of the perilymph is higher in the improved PPI (**d**) than in the conventional PPI (**a**). The signal magnitude of the endolymph is higher in the improved PEI (**e**) than in the conventional PEI (**b**). The significant endolymphatic hydrops in the left cochlea and vestibule (arrows in **c** and **f**) is more conspicuously depicted in the i-HYDROPS image (**f**) than in the conventional HYDROPS image (**c**). There is no endolymphatic hydrops in the right inner ear. (**g–i**) Enlarged view of the left inner ear with significant endolymphatic hydrops. (**g**) magnetic resonance (MR) cisternography (TR/TE = 4400/544), (**h**) HYDROPS image, (**i**) i-HYDROPS image. The markedly enlarged endolymphatic space is more conspicuous on the i-HYDROPS image than on the HYDROPS image (arrows on **g** and **i**). Note that the i-HYDROPS image is slightly noisier than the HYDROPS image.

The mean CNR value ± the standard deviation was 86.8 ± 26.5 for the HYDROPS images and 90.3 ± 29.2 for the i-HYDROPS images (*P* = 0.645). There was no significant difference between the two images, although the scan time for the i-HYDROPS images was less than half that of the HYDROPS image (Fig. 1).

For the %EL of both the cochlea and the vestibule, there were no significant differences between the two images. The average %EL of the cochlea was 11.6 ± 11.5% on the HYDROPS image and 11.4 ± 11.3% on the i-HYDROPS image (*P* = 0.87), and that of the vestibule was 22.5 ± 32.7% and 20.3 ± 31.7% respectively (*P* = 0.23). The Pearson’s correlation coefficient was 0.910 for the cochlea and 0.974 for the vestibule (*P* < 0.0001).

## Discussion

In the present study, the i-HYDROPS image had a comparable CNR to that of the HYDROPS image, even though the scan time was decreased by more than 50%. The %EL of both the cochlea and the vestibule showed a very high correlation between the two methods.

The sensitivity of the hT_2_w-3D-FLAIR image (PPI) to low concentrations of GBCA in fluid was increased by elongation of the TR.^5^ Also, the absolute value of the signal magnitude of the endolymph on the PEI was increased by elongation of the TR. This is because the difference of the null point for the endolymph without gadolinium and the gadolinium-containing perilymph was increased by elongation of the TR.^4^ The time efficiency in terms of the CNR was better with the i-HYDROPS imaging than with HYDROPS imaging. Increased time efficiency and sensitivity can be used either to reduce scan time or, potentially to reduce the dose of the contrast agent. It might be also possible to widen the appropriate scan time window for the endolymphatic hydrops evaluation, which is currently between 3–6 hours.^11^ The PPI is also expected to be a tool to evaluate the glymphatic system by analyzing the enhancement of the cerebrospinal fluid and the fluid in the perivascular space of the basal ganglia.^12^ The increased sensitivity of the PPI to low concentrations of GBCA in fluid might be useful for the evaluation of the glymphatic system.

There are some limitations to the present study. The effect of the increased refocusing flip angle in terms of the CNR was not evaluated separately in the present study. Future research comparing low and high flip angle protocols is warranted. The noise estimation by the ROI in air might not be an ideal method for images obtained using a parallel imaging technique; however, it might be acceptable for the purpose of a comparison between the two imaging protocols when the ROI is placed in an identical position on both images. The number of patients and number of observers were small. Further scan time reduction using *HYbriD of Reversed image Of MR cisternography and positive Perilymph Signal by heavily T_2_-weighted 3D-FLAIR* (HYDROPS2) type image processing^6,8^ should be evaluated in the future. Further elongation of the TR was not possible due to a limitation of the scanner. A longer TR protocol might be evaluated in the future.

## Conclusions

The newly proposed method (i.e., i-HYDROPS) allowed for a shorter scan time while providing similar CNR and %EL values compared to the conventional HYDROPS method.

## Figures and Tables

**Table 1.** Pulse sequence parameters

Sequence name	Type	Repetition time (ms)	Echo time (ms)	Inversion time (ms)	Flip angle (degree)	Section thickness/gap (mm)	Pixel size (mm)	Number of slices	Echo train length	Field of view (mm)	Matrix size	Number of excitations	Scan time (min)
MR cisternography (MRC)	SPACE with restore pulse	4400	544	NA	90/ initial 180 decrease to constant 120	1/0	0.5 × 0.5	104	173	165 × 196	324 × 384	1.8^*^	3
Heavily T_2_ weighted 3D-FLAIR (PPI)	SPACE with inversion pulse	9000	544	2250 (2050 for PEI)	90/ initial 180 decrease to constant 120	1/0	0.5 × 0.5	104 with slice oversampling of 38.5%	173	165 × 196	324 × 384	4^*^	14.3
Improved PPI	SPACE with inversion pulse	16000	544	2900 (2500 for improved PEI)	90/ constant 180	1/0	0.5 × 0.5	104 with slice oversampling of 7.7%	173	165 × 196	324 × 384	1.4^*^	7

Generalized autocalibrating partially parallel acquisitions (GRAPPA) × 2 for all sequences. All sequences utilize a frequency selective fat suppression pre-pulse; Each three dimensional (3D) slab is set in an identical axial orientation;

* Number of excitations more than one was employed not only to increase signal to noise ratio, but also to control aliasing artifact in slice encoding direction when the slab selective excitation mode was selected for SPACE sequence.^6^ PPI, positive perilymph image; PEI, positive endolymph image; SPACE, sampling perfection with application-optimized contrasts using different flip angle evolutions; FLAIR, fluid attenuated inversion recovery.
